# What Health System Challenges Should Responsible Innovation in Health Address? Insights From an International Scoping Review

**DOI:** 10.15171/ijhpm.2018.110

**Published:** 2018-11-28

**Authors:** Pascale Lehoux, Federico Roncarolo, Hudson Pacifico Silva, Antoine Boivin, Jean-Louis Denis, Réjean Hébert

**Affiliations:** ^1^Department of Health Management, Evaluation and Policy, School of Public Health, University of Montreal, Montreal, QC, Canada.; ^2^Institute of Public Health Research of University of Montreal (IRSPUM), University of Montreal, Montreal, QC, Canada.; ^3^Department of Family Medicine, University of Montreal, Montreal, QC, Canada.; ^4^Canada Research Chair on Patient and Public Partnership, Montreal, QC, Canada.; ^5^School of Public Health, University of Montreal, Montreal, QC, Canada.

**Keywords:** Health System Demand, Health Technology, Equity, Sustainability, International Analysis

## Abstract

**Background:** While responsible innovation in health (RIH) suggests that health innovations could be purposefully designed to better support health systems, little is known about the system-level challenges that it should address. The goal of this paper is thus to document what is known about health systems’ demand for innovations.

**Methods:** We searched 8 databases to perform a scoping review of the scientific literature on health system challenges published between January 2000 and April 2016. The challenges reported in the articles were classified using the dynamic health system framework. The countries where the studies had been conducted were grouped using the human development index (HDI). Frequency distributions and qualitative content analysis were performed.

**Results:** Up to 1391 challenges were extracted from 254 articles examining health systems in 99 countries. Across countries, the most frequently reported challenges pertained to: service delivery (25%), human resources (23%), and leadership and governance (21%). Our analyses indicate that innovations tend to increase challenges associated to human resources by affecting the nature and scope of their tasks, skills and responsibilities, to exacerbate service delivery issues when they are meant to be used by highly skilled providers and call for accountable governance of their dissemination, use and reimbursement. In countries with a low and medium HDI, problems arising with infrastructure, logistics and equipment were described in connection with challenges affecting procurement, supply and distribution systems. In countries with a medium and high HDI, challenges included a growing demand for drugs and new technology and the management of rising costs. Across all HDI groups, the need for flexible information technologies (IT) solutions to reach rural areas was underscored.

**Conclusion:** Highlighting challenges that are common across countries, this study suggests that RIH should aim to reduce the cost of innovation production processes and attend not only to the requirements of the immediate clinical context of use, but also to the vulnerabilities of the broader system wherein innovations are deployed. Policy-makers should translate system-level demand signals into innovation development opportunities since it is imperative to foster innovations that contribute to the success and sustainability of health systems

## Articulating the Health System Demand for Health Innovations

Responsible research and innovation (RRI) represents a policy-oriented endeavour that gained traction particularly in Europe as a result of the environmental, social and ethical concerns raised by technological developments.^1^ For von Schomberg, RRI is a “transparent, interactive process by which societal actors and innovators become mutually responsive to each other with a view to the (ethical) acceptability, sustainability and societal desirability of the innovation process and its marketable products.”^2^ Following suit to RRI, responsible innovation in health (RIH) suggests that health technologies could be designed to better support health systems around the world by foregrounding innovation development processes that are prospective, reflexive, inclusive and dynamically responsive to shifting needs and challenges.^3,4^ Considering the extent to which the current ways of developing and bringing to market new health technologies are highly capital-intensive^5-7^ and induce major inequities, RIH offers a new lens for policy-makers: it provides them with principles and tools to develop the innovations health systems need and thus proactively address equity and sustainability issues.

Since few attempts have been made to articulate what system-level challenges RIH should seek to address, the goal of this paper is to document in a structured way what is known about health systems’ demand for innovations. We performed a scoping review of the peer-reviewed scientific literature that examined the needs and challenges of health systems around the world between January 2000 and April 2016. While we reported elsewhere^8^ the key lessons and knowledge gaps that health services and policy researchers should consider, this paper digs further into the challenges specifically associated to health innovations.

The paper is comprised of 4 sections. First, we clarify the background to our study and introduce our analytical framework. Second, we describe the scoping review methodology that guided our analysis of an international corpus of 254 articles. Third, we present frequency distributions of the challenges that were reported in countries with a low, medium, high or very high Human Development Index (HDI) and examples of the challenges that are common across countries. Fourth, acknowledging that policy-makers “are faced with tough choices,”^4^ we clarify how RIH enables them to steer innovation towards more equitable and sustainable health systems.

### Innovation and the Challenges of Health Systems Around the World

Since the late 1980s, new health technologies, defined as the “application of organized knowledge and skills in the form of devices, medicines, vaccines, procedures and systems developed to solve a health problem and improve quality of lives,”^9^ have exerted growing pressure on health system financing and governance, raised important social and ethical concerns and threatened the sustainability of health systems.^10-13^ Health policy-makers increasingly voiced their concerns regarding the diffusion of new medical technologies as “the enormous challenges and needs confronting healthcare systems today make the governance of innovation extremely complex.”^4^

In response, health services and policy researchers generated knowledge on the individual, clinical and organizational barriers and facilitators that affect technology adoption patterns in primary care and university teaching hospitals.^14-16^ This literature underscores the importance for healthcare managers to foster strategic and continuous change management, to devise a broad set of integrated innovation governance strategies and to actively support the in-house production and use of evidence on the effectiveness and cost of these new technologies.^17-20^ Yet, scholars and practitioners of health technology assessment (HTA) often document the extent to which new technologies are misused or overused, emphasizing an “epidemic of waste”:

there is an inflexion point at which overused therapeutic and diagnostic technologies stop benefiting patients and only divert healthcare spending toward potentially low-benefit or unnecessary applications (so-called low value care), limiting our ability to provision consistent, broad-reaching healthcare to society.^11^

Since 2000, the unparalleled offer of new drugs, devices, procedures and information technologies (IT) provoked an important quandary for health services and policy researchers: the growing inequalities exacerbated by health innovations.^9,21^ On the one hand, poor countries can hardly afford the expensive technologies developed for rich countries as primary markets.^22^ On the other hand, access to expensive healthcare is frequently compromised in rich countries where third-party payers are struggling to adapt their cost control strategies to a rapidly expanding supply of innovations.^23^ In publicly funded systems, such strategies often aim to rationalise global health expenditures whereas, in privately funded systems, they seek to protect, maintain or increase the profit margins of health insurers.^13^ In both cases, health innovation is designed for and made available to the better off, which partly explains why Gardner and colleagues^22^ stress that “innovation systems respond best to the needs of those who can afford their outputs.” For these authors, to improve access to essential products and services in low and middle-income countries, policy-makers should support: (1) technological innovation with added societal value “to ensure availability of products that are more cost-effective than existing interventions” (eg, a frugal drill for orthopedic surgery); (2) social innovation “to ensure the distribution of essential goods and services” (eg, access based on ability to pay); and (3) adaptive innovation “to contextualize the adoption of goods and services to local settings” by involving providers and communities (eg, a local knowledge translation and exchange unit).^22^

In view of the complex policy issues raised by new health technologies, one may wonder why health services and policy researchers have not yet sought to synthesize what is known about system-level challenges that innovations should attend to in the first place. This is the research gap our paper seeks to address by consolidating the lessons that can be learned from a literature that is scattered across disciplines (eg, HTA, health economics, public management, public health, nursing, etc), geographic regions (industrialized countries, low and middle-income countries), patient groups (eg, children, women, vulnerable or marginalized groups, etc) and services (eg, mental health, primary care, etc). Beyond highlighting the problems raised by the supply of new technologies, it is important to contribute to a more explicit articulation of the demand for innovation from a health system perspective.^24^ This entails an analysis that goes beyond the individual demand from physicians and patients.

The “dynamic health system” framework of van Olmen and colleagues^25^ is particularly well suited to support a closer examination of system-level challenges. For these authors, health systems should be understood as dynamic, open systems wherein interactions and shifting equilibriums take place among 3 broad components: for adequate “service delivery” to unfold, proper “leadership and governance” should make available the right set of “resources,” which include human resources, finances, infrastructure and supplies, knowledge and information systems. These key components must also adapt to shifting population needs and contexts and align with a set of values and principles. Within their framework, innovations such as vaccines, medical devices and drugs are part of “infrastructure and supplies,” while IT solutions and knowledge-based tools such as computerized medical records, telecare or patient decision aids fall within “knowledge and information systems.”

**Figure 1:**
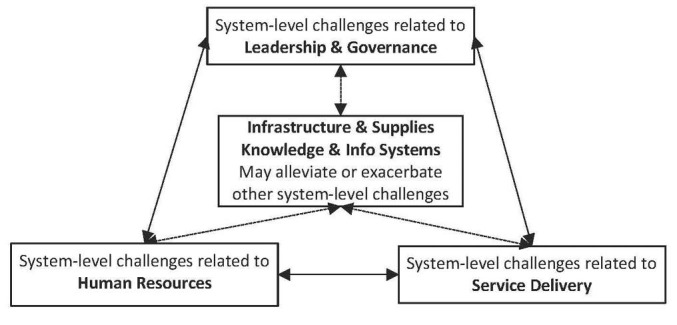
Analytical Framework.
Source: Adapted from the more exhaustive framework of van Olmen et al.^24^

Although van Olmen et al^25^ do not explicitly recognize this point, innovations may affect more than one health system component simultaneously, as illustrated by the dotted arrows in Figure 1. For instance, innovations transform service delivery by providing patients and service providers with new means to screen, diagnose and treat diseases, thereby putting pressure on human resources since their proper use may require new sets of expertise and skills.^26,27^ While certain innovations may contribute to alleviate system-level challenges, such as telehomecare applications that reduce unnecessary emergency visits,^28^ the growing complexity of other innovations may exacerbate existing challenges. For instance, though genomics technologies have been developed at a rapid pace and some at a lesser cost, their proper integration within health systems requires a host of auxiliary adaptations in terms of infrastructures, staff training, clinical guidelines, patient decision aids, etc. For Lucassen and Houlston, to meet future needs in genomics, “comprehensive resources with a far more overarching remit will need to be developed” in conjunction with the adoption of “automated machine learning, support vector machines and other technologies to create systematic and efficient mechanisms to assess the impact of variants found by genomic sequencing.”^29^ As we further explain below, RIH rather suggests to realign the purposes of technological development towards equitable and sustainable health systems.

### The Principles and Processes of Responsible Innovation in Health

In this paper, RIH is understood as a “collaborative endeavour wherein stakeholders are committed to clarify and meet a set of ethical, economic, social and environmental principles, values and requirements when they design, finance, produce, distribute, use and discard sociotechnical solutions to address the needs and challenges of health systems in a sustainable way.”^30^ The term “sociotechnical” derives from an “innovation systems lens,” which “highlights the need to create and implement both social and technological solutions.”^22^ The definition of RIH highlights the importance of supporting sustainable health systems, which are defined as: (1) *affordable* for patients, families, employers and the government acknowledging that “employers and the government ultimately rely on individuals as consumers, employees, and taxpayers for their resources”; (2) *acceptable* to “key constituents, including patients and health professionals”; and (3) *adaptable* since “health and healthcare needs are not static” and health systems “must respond adaptively to new diseases, changing demographics, scientific discoveries, and dynamic technologies in order to remain viable.”^12^

While RIH foregrounds issues that are specific to the health sector, it builds on many of the principles of RRI. For Stilgoe and colleagues,^3^ RRI should rely on 4 process-oriented principles: (1) *anticipation* of the risks and opportunities of innovation; (2) *reflexivity* towards the value systems and social practices in which innovations are embedded; (3) *inclusion* of stakeholders in order to share roles and responsibilities and democratize technological choices; and (4) *responsiveness* to unforeseen consequences that may occur along innovation trajectories and across innovation ecosystems. Building on these principles, Demers-Payette et al^4^ argued that those who develop RIH should seek to: (1) understand the various contexts of use in order to identify opportunities for innovation as well as the social, ethical and political risks that are likely to arise (anticipation); (2) harmonize the value systems and practices that govern the context in which innovations are produced (the business sector) and used (the health sector) (reflexivity); (3) integrate the views of professional and lay users and diversified publics into innovation development processes (inclusion); and (4) learn how to adapt innovative trajectories by responding to emerging views, norms, knowledge and regulatory frameworks (responsiveness).

While these principles are meant to support a prospective, reflexive, inclusive and responsive innovation development strategy, a consolidated articulation of the demand from a health system perspective is lacking. Hence, using the framework depicted in Figure 1, our study’s goal is to document and structure key elements of this demand by examining the nature, scope and implications of the system-level challenges reported in the peer-reviewed literature published between 2000 and 2016. This is a period during which many innovations and structural changes were introduced and thus yields precious insights into the challenges RIH should address. We chose not to limit our scoping review to certain groups of countries. An international analysis seems particularly timely since countries such as India, Brazil, South Africa, and China have emerged as “world leaders” in the production of innovative health products to address important domestic health needs.^22^ This resulted from a rapid consolidation and refinement of their national innovation systems, opening up a “critical window” where lessons learned around “policies that promote both public health and economic development” can be shared.^22^

## Methods

### Scoping Review Search Strategy and Article Selection Process

Scoping reviews are a specific type of review that aim to map existing literature in a broad field of interest. They are particularly well suited to rapidly describe what is known in a given research area and identifying key lessons and knowledge gaps.^31,32^

Following the steps described by Levac et al,^33^ we developed our bibliographic search strategy by identifying a set of exemplary articles (n = 43) through consultation with experts and a structured search on PubMed. This initial set of studies was used to refine our final search strategies with the help of a scientific library specialist who pretested and executed these strategies.

A total of 8 online bibliographic databases were searched in March 2016: PubMed, EMBASE, PsycINFO, International Bibliography of the Social Sciences-ProQuest (IBSS), Sociological Abstracts-ProQuest, Worldwide Political Science Abstracts-ProQuest, Public Affairs Information Service-ProQuest (PAIS), and Web of Science-SCI, SCII. All database searches were limited to a publication date between January 2000 and April 2016. To be included, papers had to: (1) have an abstract; (2) be written in English, French, or Italian; (3) be peer-reviewed; and (4) report on health system challenges or needs. “Needs” referred to the human, financial or material resources that are deemed necessary to improve and sustain the functioning of health systems and “challenges” referred to “emerging and enduring problems that destabilise the current functioning, performance or sustainability of health systems.”^8^ Articles describing specific vertical programs were excluded since they focus on a single health condition and pursue short- or medium-term objectives. After excluding duplicates, 2 reviewers independently screened the abstracts against inclusion and exclusion criteria and when necessary the full article was retrieved to resolve disagreements. All articles that met the inclusion criteria were read by one of the authors (FR) to ascertain whether they addressed our study’s aim. As per the principles of the scoping review methodology, the quality of the studies was not formally assessed.

### Collating, Summarizing and Reporting the Results

An Excel file was created to gather information related to: (1) the citation of the article (authors, title, year, affiliation of the first author); (2) details regarding the nature and scope of the article (type of article, objectives, country studied, respondents, area of interest, target population); (3) *verbatim* excerpts describing all challenges reported in the article; and (4) the category to which referred each of these challenges. This dataset thus contains challenges that were reported by investigators, not necessarily challenges that are considered more important by policy-makers or practitioners.

For each article, we extracted all the reported challenges and used a single category to classify each challenge following van Olmen and colleagues’ framework. Within each category, we developed subcategories to identify the specific challenges described by the authors. The coding strategy was refined inductively and through discussions with team members. The goal was to be able to categorize the challenges as closely to the authors’ description as possible, while relying on van Olmen and colleagues’ framework to maintain consistency.

The countries where the studies had been conducted were classified along the 2015 HDI, which combines indicators relevant to population health: life expectancy at birth, mean years of schooling and expected years of schooling, and gross national income per capita.^34^ When health systems in more than one country were examined, we only classified the articles that pertained to countries belonging to the same HDI group. For the specific purposes of this paper, we only included the articles that could be classified along the HDI. Several research team meetings were carried out during the data extraction period and the data charting form was updated and modified iteratively.

Our data analyses combined quantitative and qualitative content analysis. First, we established the frequency distributions of the challenges to identify what categories and subcategories were the most frequently reported within and across the HDI groups. Our assumption was that challenges that are frequently discussed in the literature may reflect important system-level priorities. Second, once the rows of the Excel file were sorted according to the HDI groups, we performed a qualitative analysis of the *verbatim* content extracted from the articles. This is similar to a “site–ordered matrix” analysis^35^ if one considers the HDI groups as the “sites.” This matrix enabled us to systematically look for and identify both variations and similarities across our whole dataset by comparing how authors explained the nature of the challenge being reported, its impact (if any) on other system-level challenges and its relationship to innovation. For instance, some authors argued that “innovation x” was needed to address “challenge a,” whereas others explained that “challenge b” was raised by “innovation y.”

The presentation of our findings is structured around our analytical framework (Figure 1) clarifying: what challenges were the most frequently reported in countries with a low, medium, high or very high HDI; the nature, scope and implications of the challenges related to service delivery, human resources, and leadership and governance; and the more specific challenges raised by infrastructure and supplies (eg, drugs, vaccines, surgical procedures, devices, etc.) and knowledge and information systems (eg, IT, telehealth, clinical guidelines, HTA, etc). We provide examples of challenges from different countries, while avoiding redundancies.

## Results

### An Overview of the Analyzed Articles

**Figure 2:**
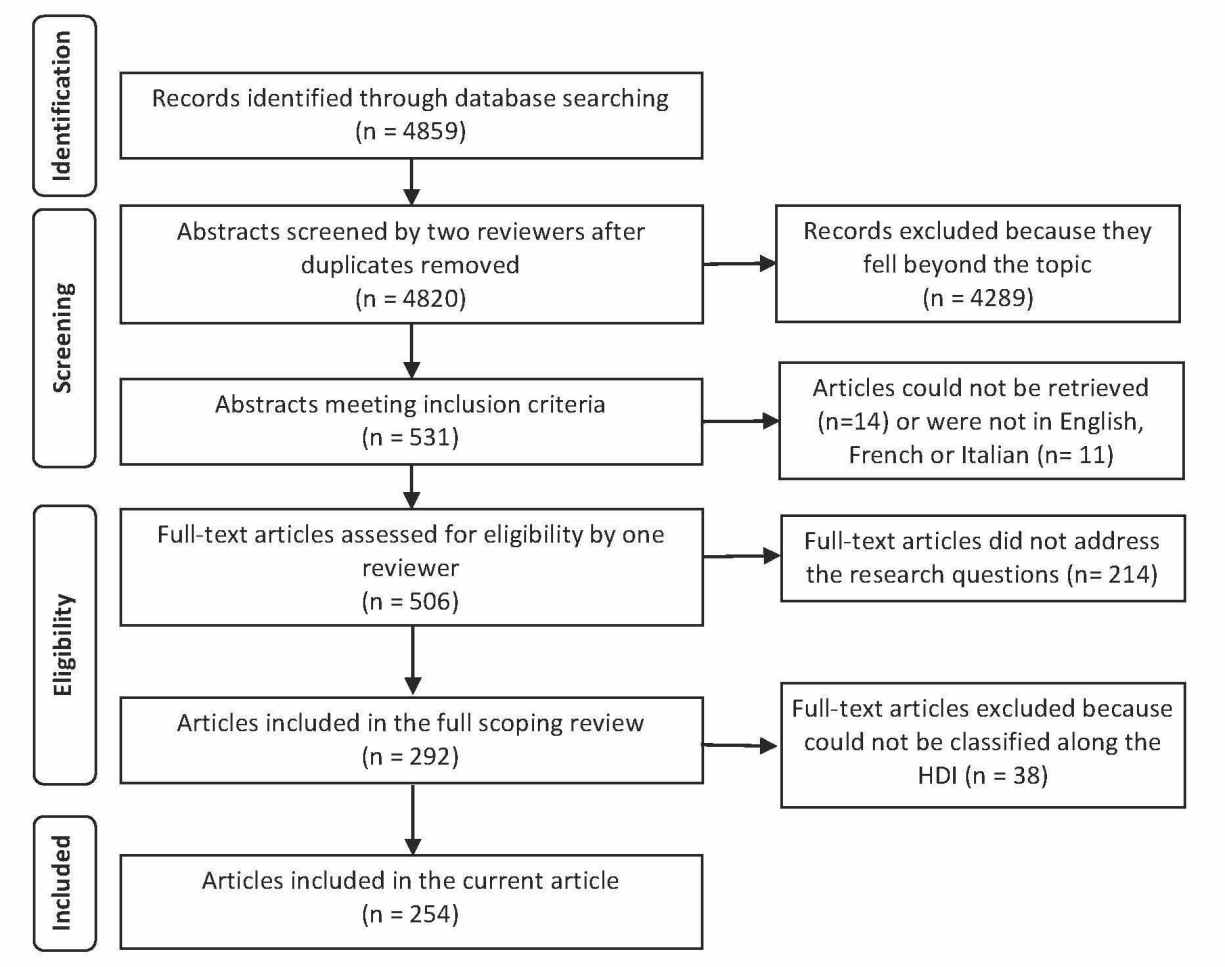
PRISMA Diagram of the Article Selection Process.

Figure 2 illustrates the article selection process. After exclusion of duplicates and the independent screening of 4820 abstracts by 2 reviewers, a total of 531 abstracts met our inclusion criteria. The full text could not be retrieved for 14 of these references and 11 were excluded because the articles were not written in English, French, or Italian. A total of 506 articles were read by one of the coauthors and 292 articles were included in the full scoping review. Among these articles, 38 could not be classified along the HDI because they either examined multiple countries that fell within different HDI categories or global challenges. The 254 articles that were included in this review reported findings pertaining to 99 countries.

More than a third of the included studies were formal empirical studies (37%) and more than a third (37%) were classified as “analyses” since they did not rely on primary research. Among the empirical studies, the majority were qualitative (63%), while 14% of them applied mixed methods. While the majority of the articles examined countries within the very high HDI category (60%), in each category there is at least one country that is over-represented when compared to the others: the United States in the very high HDI category (48%); China in the high HDI category (35%); India and South Africa in the medium HDI category (29% and 25%); and Pakistan in the low HDI category (21%). Table 1 provides a summary of the characteristics shared by countries falling within each HDI group.

**Table 1:** Indicators Related the HDI Groups

	**Low HDI** **<0.550**	**Medium HDI** **0.550–0.700**	**High HDI** **0.700–0.799**	**Very High HDI** **≥0.800**
Life expectancy at birth (y)	59.8	66.6	74.7	79.7
Expected years of schooling	9.4	11.4	14.0	16.1
Mean years of schooling	4.3	6.8	9.5	11.5
Gross national income per capita (PPP $)	3507	6302	14 524	41 491
Main regions	Sub-Saharan Africa	South Asia, Arab States	Central Asia, Latin America and The Caribbean, East Asia and The Pacific	Europe, North America, Other countries^a^

Abbreviation: HDI, human development index. ^a^ Australia, Singapore, Japan, South Korea, Israel, Brunei, Cyprus, Qatar, Chile, Saudi Arabia, United Arab Emirates, Argentina, Bahrain, Russia, Kuwait. Source: UNDP.^36^

### System-Level Challenges Reported Across Countries

A total of 1391 challenges were extracted from the articles. Table 2 provides the distribution of these challenges along the HDI and van Olmen and colleagues’ 8 broad categories of challenges. It shows that the more frequently examined challenges across the 4 HDI groups belong to 3 categories: health service delivery (25%), human resources (23%), and leadership and governance (21%). It also indicates that not much research reported on challenges falling within the remaining categories: finances (8%), infrastructure and supplies (8%), knowledge and information (7%), principles and values (6%), and population and context (3%).

**Table 2:** Distribution of the System-Level Challenges Reported in the Scientific Articles (n = 254) That Were Classified Along the HDI

**Category of challenge**	**Low HDI**		**Medium HDI**		**High HDI**		**Very High HDI**		**Total**	
	**No.**	**%**	**No.**	**%**	**No.**	**%**	**No.**	**%**	**No.**	**%**
Health Services Delivery	33	15	33	18	80	26	195	29	341	25
Human resources	68	30	52	28	69	22	124	18	313	23
Leadership and governance	50	22	50	27	61	20	131	20	292	21
Finances	14	6	10	5	29	9	63	9	116	8
Infrastructure and supplies	26	11	17	9	25	8	44	7	112	8
Knowledge and information	25	11	10	5	12	4	49	7	96	7
Principles and values	4	2	9	5	26	8	43	6	82	6
Population and context	7	3	5	3	5	2	22	3	39	3
Total (challenges)	227	100	186	100	307	100	671	100	1391	100

Abbreviation: HDI, human development index. Note: The 2 shades of grey respectively indicate the first and second most frequently reported challenges.

When examining variations across the 4 HDI groups, one may observe that 6 challenge categories were reported in roughly comparable proportions. Only service delivery and human resources present a different pattern where up to 30% of the challenges reported in countries with a low HDI referred to human resources and up to 29% of the challenges reported in countries with a very high HDI pertained to service delivery. This may reflect the fact that more mature health systems manage a larger supply of services whereas less mature health systems depend upon the development of a qualified workforce.

### Challenges Related to Service Delivery, Human Resources, and Leadership and Governance

Table 3 indicates the distribution of the specific challenges falling within 3 challenge categories. Those pertaining to service delivery include services delivered for the prevention, promotion and treatment of acute and chronic conditions. It is striking that across the 4 HDI groups the most often reported challenges within this category referred to access (32%). In countries with a low and medium HDI, the second most frequently reported challenges pertained to delivery, quality and safety (respectively, 27% and 27%). In countries with a high HDI, the second most often reported challenges pertained to referral systems and vertical integration (16%), whereas in countries with a very high HDI they pertained to cultural and linguistic barriers and the difficulty of obtaining information about services (19%).

**Table 3:** Distribution of the Challenges Specific to Service Delivery, to Human Resources, and to Leadership and Governance

	**Low HDI**		**Medium HDI**		**High HDI**		**Very High HDI**		**Total**	
	**No.**	**%**	**No.**	**%**	**No.**	**%**	**No.**	**%**	**No.**	**%**
**Service Delivery**										
Access	7	21	9	27	28	35	64	33	108	32
Delivery, quality, and safety	9	27	9	27	9	11	33	17	60	18
Info, cultural, and linguistic barriers	5	15	4	12	7	9	38	19	54	16
Continuity of care and prevention	5	15	1	3	10	13	25	13	41	12
Referral systems and vertical integration	4	12	6	18	13	16	9	5	32	9
Overuse and waste of resources	1	3	3	9	9	11	13	7	26	8
Waiting times	2	6	1	3	4	5	13	7	20	6
Total (challenges)	33	100	33	100	80	100	195	100	341	100
**Human Resources**										
Staff distribution and sufficiency	21	31	17	33	20	29	43	35	101	32
Retention, motivations, and incentives	14	21	8	15	21	30	21	17	64	20
Competency and monitoring	18	26	8	15	15	22	17	14	58	19
Education and training	10	15	8	15	8	12	15	12	41	13
Provider-patient communication	0	0	7	13	2	3	20	16	29	9
Workload and safety	5	7	4	8	3	4	8	6	20	6
Total (challenges)	68	100	52	100	69	100	124	100	313	100
**Leadership and Governance**										
Strategic policies and governance	16	32	15	30	18	30	41	31	90	31
Oversight and accountability	16	32	15	30	19	31	27	21	77	26
Horizontal coordination	10	20	10	20	13	21	41	31	74	25
Patient and community engagement	5	10	5	10	4	7	14	11	28	10
Regulations	3	6	5	10	7	11	8	6	23	8
Total (challenges)	50	100	50	100	61	100	131	100	292	100

Abbreviation: HDI, human development index. Note: The 2 shades of grey respectively indicate the first and second most frequently reported challenges.

Our analyses identified many examples where access to services was compromised because of geographic, financial or sociocultural barriers (see Supplementary file 1). For example, in the Mongolian rural areas (high HDI) described by Lindskog, the combined effect of these barriers impedes “herder families’ ability to access basic healthcare services and life-saving medical treatment” because of “long distances to health services and lack of transportation; lack of money for health insurance; run-down hospitals, unqualified health personnel and lack of medical equipment.”^37^ The need to ensure proper access to services increases when caring for vulnerable populations. In the United States, Kugel and Zoroweste recommended to support “continuity of care and tracking for the mobile poor with the use of new technologies (cell phones, electronic health records, Internet, virtual case management) to ensure follow-up of abnormal tests and continuity of treatment regimens for mobile patients.”^38^ For Patterson and Hovey,^39^ though “it is not surprising” that most American primary physicians would be unfamiliar with the full range of equipment and therapies available for the large number of pediatric chronic health conditions, it limits their ability to properly inform parents and impedes continuity of care.

As Table 3 indicates, the distribution of the challenges related to human resources, which include health service providers, health managers and support workers in private and public health and social care organizations, varies slightly along the HDI: the most frequently reported challenges referred to staff distribution and sufficiency (respectively, 31%, 33%, 29%, and 35%), staff retention, motivations and incentives (respectively, 21%, 15%, 30%, and 17%), and staff competency and monitoring (respectively, 26%, 15%, 22%, and 14%).

While the availability of staff is dependent upon a country’s capacity to train, recruit, retain and support qualified personnel, innovations may exacerbate these challenges. In a low HDI country like Malawi, Lemay et al^40^ underscored that “a common challenge mentioned by community health workers is the delay in receiving up-to-date information and the necessary materials for their jobs.” Effective health workers have to master the technical knowledge and skills required to provide high quality care, but they must also possess interpersonal skills to engage in patient-centred care. The literature we analyzed indicates that health innovations directly affect the former and may indirectly impact the latter. For instance, in Russia (high HDI), Sheman and Shishkin^41^ observed that “district doctors are not adequately trained in modern medical technologies, and as a result, there is a growing lack of trust in them, and more and more patients want to receive even simple medical care in hospitals.” Heterogeneous work environments also modulate the competency of human resources. Canadian home care providers had to adapt to “inadequate or unfamiliar technology”^42^ that reduced their ability to properly respond to rural residents’ needs.

The leadership and governance category refers to the government’s various roles in health policy ranging from policy implementation to community engagement, including coordination with private and public organizations whose activities impact population health (see Table 3). Across the 4 HDI groups, the most often reported challenges referred to strategic policies and governance (31%), oversight and accountability (26%), and horizontal coordination (25%).

Similar governance issues rested with the development of high-level comprehensive health policies that appropriately regulate the use of innovations. The lack of policy frameworks in Papua New Guinea (low HDI) pushed service providers to make rationing decisions “on a day-to-day basis in direct clinical care.”^43^ Melo and Sequeiros^44^ underscored that expanding “appropriate and useful genetic testing” services in the Brazilian (high HDI) public health system would require “adding effective HTA, ethical meaning, and social responsibility to the provision of medical genetics services.” In Saudi Arabia (very high HDI), Al-Sharqi and Abdullah^45^ described how the regulation of healthcare insurance coverage poses equity challenges since regional disparities are evident “in resources and infrastructure, medical technology and equipment, professional expertise, the quality of care, and standards of practices.”

Effective implementation of health policies requires strong horizontal coordination with organizations that operate outside the health sector. Chopra et al^46^ argued that effective multisectoral policies must be implemented in South Africa (medium HDI) “to target alcohol control, sexual violence and inequality, diet and physical activity, hygiene and sanitation, and sustainable development.” The capacity of the government to play such a broad and strong role was appraised differently across countries. For Warsame et al,^47^ “poor governance in the Somali Health System [low HDI] has been symptomatic of the breakdown in the wider governance of the country, which has been in a state of extreme fragility for more than 2 decades” and, as a result, capacity for stewardship was limited.

While the impacts of a poor regulation of pharmaceuticals took different forms across countries (see Supplementary file 1), the importance of proper oversight and accountability mechanisms was similarly underscored. In Somalia (low HDI), assessing the quality and authenticity of the drugs brought into circulation by the “largely unregulated” pharmaceutical industry remains difficult.^47^ For Yip et al,^48^ governance problems in China (high HDI) are compounded by “collusion between providers and the pharmaceutical sector” as certain hospitals receive “kickbacks from drug companies for prescribing their products” and doctors’ “bonuses are often tied to these kickbacks.”

Overall, innovations may increase system-level challenges that are associated to human resources by affecting the nature and scope of their tasks, skills and responsibilities. While they raise patients’ expectations, innovations exacerbate service access issues when they can only be used by highly skilled providers, when their installation and maintenance require sophisticated resources or when their integration within service delivery affects continuity of care or prohibits their system-wide dissemination. As a result, the need for health policies that manage expectations and comprehensively and accountably regulate the dissemination, use and reimbursement of innovations becomes salient.

### Challenges Related to Infrastructure and Supplies, and Knowledge and Information Systems

Table 4 indicates the distribution of the challenges falling within the last 2 categories. The infrastructure and supplies category refers to the ‘hardware’ of health systems. In principle, to support proper care delivery, infrastructures should be accessible by patients, well equipped, well maintained, adapted to population needs and include a reliable supply system for technologies, drugs and other commodities. Challenges pertaining to infrastructure, logistics and equipment were the most frequently reported overall (31%). In countries with a low and medium HDI, the most frequently reported challenges had to do with infrastructure, logistics and equipment (respectively, 35% and 41%) and procurement, supply and distribution systems (respectively, 46% and 24%). In contrast, in countries with a high and very high HDI, challenges related to the cost of drugs and technologies (respectively, 24% and 27%) and to the growing need or demand for drugs and technologies (respectively, 24% and 27%) were more frequently reported.

**Table 4:** Distribution of the Challenges Specific to Infrastructure and Supplies, and to Knowledge and Information Systems

	**Low HDI**		**Medium HDI**		**High HDI**		**Very High HDI**		**Total**	
	**No.**	**%**	**No.**	**%**	**No.**	**%**	**No.**	**%**	**No.**	**%**
**Infrastructure and Supplies**											
Infrastructure, logistics, and equipment	9	35	7	41	8	32	11	25	35	31
Procurement, supply and distribution systems	12	46	4	24	4	16	5	11	25	22
Costs of drugs and tech	1	4	3	18	6	24	12	27	22	20
Individual need/demand for drugs and tech	0	0	2	12	6	24	12	27	20	18
Quality and efficacy	4	16	1	6	1	4	4	9	10	8
Total (challenges)	26	100	17	100	25	100	44	100	112	100
**Knowledge and Information Systems**										
Information production	9	36	3	30	2	17	11	23	25	26
Information availability	6	24	2	20	3	25	12	24	23	24
Information analysis and sharing	5	20	1	10	3	25	12	24	21	22
Information use	4	16	2	20	3	25	8	16	17	18
Information systems	1	4	2	20	1	8	6	12	10	10
Total (challenges)	25	100	10	100	12	100	49	100	96	100

Abbreviation: HDI, human development index. Note: The 2 shades of grey respectively indicate the first and second most frequently reported challenges.

Problems related to infrastructure, logistics and equipment were described as having diverse consequences (see Supplementary file 1), but their sources were intimately linked to the challenges raised by the transportation and distribution of goods and supplies. In Ethiopia (low HDI), Cowan et al^49^ observed that “pharmacists raised concerns regarding medication inventory, describing shortages of first-line tuberculosis medications” in several sites and noted “regularly receiving medications delivered near or past expiration.” In Papua New Guinea (low HDI), an unreliable supply of drugs meant both shortages and excesses: “there is little stock control and, as a result, facilities are short of some items and carry excesses of others.”^43^ The need to adapt and evolve one’s infrastructure in order to adequately address current epidemiologic shifts was stressed by Morhason-Bello and colleagues^50^ for whom sub-Saharan countries need to diagnose and treat cancer. It should be pointed out that infrastructure issues were also reported in countries with a very high HDI. Cristofalo and colleagues^51^ studied 2 American community health centers in the “urban core of Seattle with a rich mix of ethnic and racial diversity and socioeconomic disadvantage” and found “physical facility issues, such as archaic phone systems, overcrowded waiting rooms, and lack of offices for assessments and examinations.”

In countries with a high and very high HDI, the individual demand for drugs and new technology was typically discussed in light of its financial impact. Merican et al^52^ underscored that “demands for new expensive medications such as anti-hypertensive drugs and new types of high cost services such as transplants and implants and ICT based services, will have a significant impact on the limited health resources available” in Malaysia (high HDI). In Romania (high HDI), such demand was linked to a better-informed citizenry: “the increase of the awareness of the citizens-patients in relation to the technological development of the diagnostic and therapeutic methodologies will contribute to an increase of the expectations and demands for top medical services.”^53^ For Peiro and Barrubes,^54^ the improper use of health technology unduly increases health spending in Spain (very high HDI) because of “an increase in indications for inappropriate medical and surgical procedures, unnecessary pharmaceutical prescriptions, or an increase in the population targeted for treatment.”

As Table 4 indicates, the distribution of challenges associated to knowledge and information systems varies moderately across countries, with the most frequently reported referring to the ability to produce information (26%), to make it available to key users (24%), and to analyze and share it adequately (22%).

The importance of producing knowledge and supporting its use across various health system components in order to foster evidence-based management and practice was underscored across all HDI groups. For Lemay et al,^40^ managers and providers in Malawi (low HDI) would benefit from “evidence-based technical and clinical information” if it had “local relevance” in terms of context and language and included “easy-to-use materials and tools.” According to Ruff and colleagues,^55^ information systems would help to better anticipate and adapt to shifting population needs in South Africa (medium HDI) by supporting “proactive population management.” The proper adoption of new technology was associated to the need to build capacity in HTA and support evidence-based clinical practices. For instance, Davari et al^56^ stressed that Iran (high HDI) had “no systematic method of evaluation and guideline creation for the utilization of new and high cost technologies.” In a vast country like Canada (very high HDI), McGrail et al called for better knowledge sharing mechanisms across provinces in order to scale up innovations proven effective because “too often” the results of “experiments and innovations stay local.”^57^

Several authors discussed whether IT solutions could support the delivery of services. To support mental health services in low- and middle-income countries, Hanlon and colleagues^58^ suggested that “peer support or remote supervision using telemedicine” need to be considered. In the US, Groman and Rubin^59^ wondered why “despite recent investments” in IT solutions, infrastructure and “interoperable information technology to support clinical and administrative processes” were still not available. Charlton et al^28^ who examined American teleoncology programs for rural cancer patients argued that: “broadband internet, necessary for the use of modern day, high-quality audiovisual conferencing, is less available in rural areas, with residents being more likely to report not having broadband access because it is not available where they live.”

Overall, problems arising with infrastructure, logistics and equipment were described in countries with a low and medium HDI in close connection with local procurement, supply and distribution systems. In countries with a medium and high HDI, challenges included a growing demand for drugs and new technology and the difficulty of managing rising costs. Challenges related to knowledge and information systems highlight the need for an integrated strategy that fosters learning across various health system components and mobilizes IT solutions sufficiently flexible to reach remote and rural areas.

## Discussion

This paper contributes to fill an important research gap by providing an explicit articulation of the system-level demand for health innovation. While health services and policy scholars frequently underscore that health technology constitutes an important cost-driver that needs to be better managed,^11,13,60^ it less often analyzes the ways in which innovations may better support health systems. Our goal was thus to synthesize and structure the lessons that can be learned from an international peer-reviewed literature that is scattered across disciplines, geographic regions, patient groups and types of services, but which contains rich information about the relationships between system-level challenges and health innovations. Our scoping review contributes to current knowledge by generating novel policy insights into the system-level challenges that RIH should address.

### What System-Level Challenges Should RIH Address

RIH “not only calls for the involvement of multiple stakeholders, including the publics, but it also argues in favour of a deliberate and continuous *ex ante* consideration of what is collectively expected from innovation.”^61^ Through anticipatory, reflexive, inclusive and responsive innovation development processes, RIH could enable policy-makers to proactively address health system equity and sustainability by steering innovation towards these important goals.^4,62^ Our scoping review brings to the fore 5 cross-cutting issues for RIH to address.

First, our findings indicate the prominence of access issues across all HDI groups. This is a strong signal for those who develop frugal innovations or community-based solutions. Access issues may arise when affordability, acceptability or geographic accessibility of services is compromised.^28,37,51,63,64^ This is exacerbated by the development of sophisticated and expensive technologies designed to be used by highly skilled providers operating in urban centers.^44,65^ RIH could address such challenges by providing solutions with increased affordability, which may result from optimized production processes and/or lower maintenance needs. Aiming to reduce geographical barriers, several IT solutions were deployed throughout the past decades but sometimes failed to define how services needed to be concurrently reorganized.^15^ RIH could address such barriers by providing solutions adapted to local providers’ needs and constraints, acknowledging that increased usability would support their scalability in remote or resource-poor settings as well as appropriateness and continuity of care.^66^ Our findings also underscore that the need to ensure proper access to services increases when caring for vulnerable populations.^38,42^

Second, RIH should examine whether an innovation may increase system-level challenges that are associated to human resources by affecting the nature and scope of their tasks, skills and responsibilities. Considering that health workers with the right skill-mix should be available where and when needed,^27^ RIH should anticipate the intensity of the training an innovation may require and how its adoption by providers can be supported by their managers. Innovations that require less training or training that can be offered locally by peers should be prioritized. Our findings show that patients’ views about primary care staff and what good care entails may increase pressures on specialized care staff. Hence, RIH could support deliberate attempts to reinforce primary care and meaningfully inform their patients and the publics about screening or diagnostic tests that are known to be useless and about what makes a population healthy.^20^

Third, RIH should consider how existing economic incentives affect the governance of privately- and publicly-funded health systems.^45,67,68^ In order to reduce health inequalities, the ability to benefit from RIH should not vary according to one’s socioeconomic status, social position or capabilities. While the strengthening of governmental capacity is beyond the scope of RIH, our findings suggest that RIH should acknowledge that when oversight and transparency are compromised in a given health system, health innovations may easily become valuable commodities in informal or corrupted markets.^48,68,69^ One key challenge for RIH is indeed to anticipate and cope with a complex set of regulatory frameworks and market dynamics. Innovation scholars, policy-makers and practitioners should explore together various scenarios to ensure that proper mitigation means are in place for handling the ethical, social and legal issues that innovations raise.

Fourth, our findings highlight that infrastructural capacities and distribution systems need to be concurrently considered. There are many similarities in the way problems with infrastructure and supplies are described in countries with a low and medium HDI, ie, in close connection with the procurement, supply and distribution of drugs, vaccines and equipment. Yet, such challenges are found in rural and remote areas across all HDI groups. As such, RIH should carefully attend not only to the requirements of the immediate clinical context use, but also to the vulnerabilities of the broader context in which health innovations are deployed.^37^ In countries with a medium and high HDI, the challenges most frequently reported include a growing demand for drugs and new technology in parallel with rising costs. To address such challenges that are also present in countries with a low HDI, RIH could adopt frugal innovation principles in order to optimize the fit between an innovation’s performance level in terms of functionalities, robustness, durability, etc. and its context of use.^23^ RIH should also forestall business models that are mainly geared at generating financial returns to shareholders.^6,7^ Health services and policy researchers as well as policy-makers should pay attention to alternative business models, which include, for instance, social enterprises that reinvest their profits in their mission, adopt redistributive pricing schemes or make an innovation freely usable or exploitable by others.

Finally, our findings stress the importance of wisely tapping into IT-based solutions and supporting evidence-based management and practice. This requires considering the links between the way information is produced, “packaged” and then used. By inclusively engaging managers, clinicians and communities, RIH can inform the development of knowledge-based tools and IT solutions (eg, collaborative platforms, patient- and caregiver-oriented tools) whose local adaptation can be scaled up across remote and rural areas. Our findings show that the need to consider both the broader environment in which health innovations are deployed and their specific contexts of use also applies to IT-based services.^28,40^ This is compatible with the increasing willingness of innovative companies to “promote open standards, data exchange and interoperability in ways that facilitate collaboration across suppliers and increase potential for widespread adoption,” thereby engaging in “long-term strategic partnerships” with health and social care organizations.^66^

### Policy Recommendations

For Greenhalgh et al, current “incentive and regulatory mechanisms are not supporting or rewarding the public goods” that health systems need.^66^ This is one of the reasons why a stronger command of system-level challenges provides innovation policy-makers and health policy-makers with precious guidance. While we acknowledge important limits to RIH,^70^ its framework provides these policy-makers with principles that could facilitate coordinated action to better support the creation and adoption of innovations that bring more value to health systems (see Box 1).

**Box 1. Policy Initiatives to Better Align Health Innovation With System- Level Challenges**

**At an early stage of innovation development**

- Goal: To proactively address the equity and sustainability challenges of health systems.
- Health policy-makers should translate system-level demand “signals” into innovation development opportunities that can be prioritized by innovation policy-makers.
- Innovation policy-makers should value, finance and reward technology-based entrepreneurial activities that closely overlap with the challenges of health systems.

**
Throughout the dissemination and use of innovations
**

- Goal: To foster an inclusive international dialogue on the purposes and means of health innovation.
- Industrialized countries should acknowledge the growing innovation development capacities of emerging economies.
- Recurrent policy-oriented forums, at the local, regional, national and international levels, where researchers, practitioners and policy-makers can share lessons about innovations that better respond to system-level challenges should be established.

**
In view of the subsequent cycle of innovation and health policy development
**

- Goal: To use a well-informed system-level demand as a continuous lever for innovation.
- Innovation and health policy-makers should remain responsive to the evolving needs and expectations of their populations.
- They should align their innovation policy actions along a set of values and principles that are consistent with the goals of health systems.

First and foremost, health policy-makers should contribute to the translation of system-level demand signals into innovation development opportunities that can be prioritized by innovation policy-makers.^10^ The latter are responsible for various policies (eg, R&D tax credits, economic development strategies, sectorial priorities) that condition, upstream, the kinds of innovation that will later be “pushed” towards health systems.^24^ The technology-based entrepreneurial activities that are valued, financed and rewarded by innovation policy-makers have to closely overlap with system-level needs and challenges (eg, IT-mediated community care capacity building).^6,71^ This is particularly important if health policy-makers want to proactively address the equity and sustainability challenges health systems around the world are facing.

Second, it is important to acknowledge that countries outside the Organization for Economic Cooperation and Development (OECD) are active in health innovation development. India, China, Brazil, and South Africa are increasingly producing novel scientific and technological products.^22^ One may believe that because these countries have very large domestic markets as well as rapidly shifting population health needs, they are well positioned to engage in the development of RIH. Thus, international policy-oriented forums where researchers and policy-makers can regularly learn from each other and share lessons about innovations that better respond to system-level challenges should be encouraged.

Third, as underscored by van Olmen et al^25^, to generate their expected outcomes and improve health, health systems have to adapt to shifting population needs and contexts while aligning their priorities along a set of values and principles. By emphasizing reflexivity and responsiveness in innovation governance mechanisms, RIH could explicitly support and account for such alignment. Increasingly, health policy-makers have to respond to rising patients’ expectations, increasingly pluralistic societies and complex reforms that include an effective regulation of the private sector. Since an important tension between the call to reduce disparities in access to services and the control of health spending growth was frequently underscored in our corpus of articles, a more explicit articulation of the system-level demand for innovation may prove a powerful lever for innovation.

## Limitations

To our knowledge, this is the first attempt at articulating the health system demand for health innovations by carefully taking stock of the system-level challenges that have been reported in the peer-reviewed international literature. The key weaknesses of our scoping review should nonetheless be kept in mind. First, our findings reflect the results of our search strategy as well as the challenges that various investigators have chosen to examine and describe since 2000, not necessarily the challenges that are the most important to study or that prove the most important in practice. While it is reasonable to presume that investigators are influenced by the priorities identified by healthcare organizations, research funding agencies, managers, practitioners or patients, the absence of environmental challenges is intriguing in view of the carbon footprint of healthcare that is far from negligible.^62^ Second, although the timeframe we defined for our bibliographic search strategy was particularly relevant to capture a period marked by the emergence of many innovations, it is possible that some of the challenges identified in specific countries have been solved since their reporting. This is one of the reasons why we focused our analyses on the challenges that were recurrent across countries. Third, because the articles we reviewed often focused on a particular component of a broader national or regional health system (eg, services for veterans, indigenous communities, etc), we did not classify the types of health system being studied (for instance, the Beveridge, Bismarck, national health insurance and out-of-pocket systems). Lastly, the language restrictions we applied contributed to the under-representation of certain countries in our corpus of articles (eg, publications in Portuguese, Spanish, Japanese, German, etc).

## Concluding Remarks

Since the late 1980s, new health technologies not only increased global inequalities, but they also undermined the sustainability of health systems in rich and poor countries alike. For Fineberg,^12^ successful health systems are characterized by: (1) healthy people, that is “a population that attains the highest level of health possible”; (2) superior care, which entails “effective, safe, timely, patient-centered, equitable, and efficient” care; and (3) fairness, which implies that services are provided “without discrimination or disparities to all individuals and families, regardless of age, group identity, or place” and that the “health professionals, institutions, and businesses supporting and delivering care” are treated fairly within the health system. Along similar lines, we believe that, over the next decades, it will be imperative to implement policy mechanisms that can support the development, financing and use of innovations that do not compromise but rather contribute to the success and sustainability of health systems. The findings of this scoping review indicate that RIH should be steered towards the following key aims: (1) to reduce service access barriers (financial, geographic, cultural); (2) to match the needs, tasks, skills and responsibilities of health and social care providers; (3) to mitigate economic and political vulnerabilities affecting health system governance; (4) to reduce urban-rural disparities resulting from infrastructural capacities and the distribution of goods and supplies across regions; and (5) to scale up knowledge- and IT-based tools that are adapted to their local context of use.

## Acknowledgments

This study is part of a broader research program on RIH, which is led by the corresponding author. We would like to thank members of our research team who provided us with insightful comments throughout the data analysis and writing process: Jeremy Bouchez, Dominique Grimard, Renata Pozzelli, Lysanne Rivard, and Patrick Vachon. Elsury Pérez Isaza contributed to the scoping review article selection process.

## Ethical issues

Not applicable.

## Competing interests

Authors declare that they have no competing interests.

## Authors’ contributions

PL conceived of the study design and wrote the first draft of the manuscript. FR read all the articles included in the scoping review and analyzed their content. HPS, AB, JLD, and RH made a substantial contribution to the study. They revised the manuscript critically for important content and approved the final version.

## Funding

This research was funded by an operating grant from the Canadian Institutes of Health Research (CIHR; FDN-143294). PL holds the University of Montreal Chair on Responsible Innovation in Health (2015-2018). JLD holds a Canada Research Chair on Design and Adaptation of Health Systems. Our research group infrastructure is supported by the *Fonds de la recherche en santé du Québec* (FRQS).

## Authors’ affiliations

^1^Department of Health Management, Evaluation and Policy, School of Public Health, University of Montreal, Montreal, QC, Canada. ^2^Institute of Public Health Research of University of Montreal (IRSPUM), University of Montreal, Montreal, QC, Canada. ^3^Department of Family Medicine, University of Montreal, Montreal, QC, Canada. ^4^Canada Research Chair on Patient and Public Partnership, Montreal, QC, Canada. ^5^School of Public Health, University of Montreal, Montreal, QC, Canada.

## Supplementary files

Supplementary file 1 contains Table S1.

## Key messages

**Implications for policy makers**

- Equity and sustainability challenges of health systems should be proactively addressed.
- Health policy-makers should translate system-level demand “signals” into innovation development opportunities.
- Innovation policy-makers should reward technology-based entrepreneurial activities that closely overlap with the challenges of health systems.
- International policy-oriented forums should share lessons about innovations that better respond to system-level challenges.

**Implications for public**

The publics should contribute to the articulation of system-level needs and challenges. They should also be informed about the way equity and sustainability challenges are being addressed.

## References

[1] van Oudheusden M (2014). Where are the politics in responsible innovation? European governance, technology assessments, and beyond. Journal of Responsible Innovation.

[2] von Schomberg R (2013). A Vision of Responsible Research and Innovation In: Owen R, Bessant J, Heintz M, eds Responsible Innovation: Managing the Responsible Emergence of Science and Innovation in Society. Wiley.

[3] Stilgoe J, Owen R, Macnaghten P (2013). Developing a framework for responsible innovation. Res Policy.

[4] Demers-Payette O, Lehoux P, Daudelin G (2016). Responsible research and innovation: a productive model for the future of medical innovation. Journal of Responsible Innovation.

[5] Hirsch-Kreinsen H (2011). Financial market and technological innovation. Industry and Innovation.

[6] Lehoux P, Miller FA, Daudelin G, Urbach DR (2015). How venture capitalists decide which new medical technologies come to exist. Sci Public Policy.

[7] Robinson JC (2015). Biomedical innovation in the era of health care spending constraints. Health Aff (Millwood).

[8] Roncarolo F, Boivin A, Denis JL, Hebert R, Lehoux P (2017). What do we know about the needs and challenges of health systems? A scoping review of the international literature. BMC Health Serv Res.

[9] World Health Organization (WHO). Medical devices: managing the mismatch: an outcome of the priority medical devices project. Geneva: WHO; 2010.

[10] Gelijns AC, Russo MJ, Hong KN, Brown LD, Ascheim DD, Moskowitz AJ (2013). Dynamics of device innovation: implications for assessing value. Int J Technol Assess Health Care.

[11] Lawler PR, Norheim OF (2015). Clinical Practice guidelines as instruments for sound health care priority setting. Am J Cardiol.

[12] Fineberg HV (2012). A successful and sustainable health system—how to get there from here. N Engl J Med.

[13] Marchildon G (2013). Canada: Health system review. Health Syst Transit.

[14] Adler-Milstein J, Jha AK (2012). Sharing clinical data electronically: a critical challenge for fixing the health care system. JAMA.

[15] Greenhalgh T, Robert G, Macfarlane F, Bate P, Kyriakidou O, Peacock R (2005). Storylines of research in diffusion of innovation: a meta-narrative approach to systematic review. Soc Sci Med.

[16] Dixon-Woods M, Amalberti R, Goodman S, Bergman B, Glasziou P (2011). Problems and promises of innovation: why healthcare needs to rethink its love/hate relationship with the new. BMJ Qual Saf.

[17] Henshall C, Schuller T (2013). Health technology assessment, value-based decision making, and innovation. Int J Technol Assess Health Care.

[18] Brown AD, Alikhan LM, Sandoval GA, Seeman N, Baker GR, Pink GH (2005). Acute care hospital strategic priorities: perceptions of challenges, control, competition and collaboration in Ontario’s evolving healthcare system. Healthc Q.

[19] DiSesa VJ, Kaiser LR (2015). What’s in a name? The necessary transformation of the academic medical center in the era of population health and accountable care. Acad Med.

[20] Millar J, Bruce T, Cheng SM, Masse R, McKeown D (2013). Is public health ready to participate in the transformation of the healthcare system?. Healthc Pap.

[21] Jacobs M, El-Sadr WM (2012). Health systems and health equity: the challenge of the decade. Glob Public Health.

[22] Gardner CA, Acharya T, Yach D (2007). Technological and social innovation: a unifying new paradigm for global health. Health Aff (Millwood).

[23] Garber S, Gates SM, Keeler EB (2014). Garber S, Gates SM, Keeler EB, et alRedirecting Innovation in UShealth care: options to decrease spending and increase value. Rand Health Q.

[24] Lehoux P, Williams-Jones B, Miller F, Urbach D, Tailliez S (2008). What leads to better health care innovation? Arguments for an integrated policy-oriented research agenda. J Health Serv Res Policy.

[25] van Olmen J, Criel B, Van Damme W (2010). Analysing health systems to make them stronger. Studies in Health Services Organisation & Policy.

[26] Wallace J, McNally S, Richmond J, Hajarizadeh B, Pitts M (2012). Challenges to the effective delivery of health care to people with chronic hepatitis B in Australia. Sex Health.

[27] Wang HH, Wang JJ, Zhou ZH, Wang XW, Xu L (2013). General practice education and training in southern China: recent development and ongoing challenges under the health care reform. Malays Fam Physician.

[28] Charlton M, Schlichting J, Chioreso C, Ward M, Vikas P (2015). Challenges of rural cancer care in the United States. Oncology (Williston Park).

[29] Lucassen A, Houlston RS (2014). The challenges of genome analysis in the health care setting. Genes (Basel).

[30] Pacifico Silva H, Lehoux P, Miller FA, Denis JL (2018). Introducing responsible innovation in health: a policy-oriented framework. Health Res Policy Syst.

[31] Arksey H, O’Malley L (2005). Scoping studies: towards a methodological framework. Int J Soc Res Methodol.

[32] Rumrill PD, Fitzgerald SM, Merchant WR (2010). Using scoping literature reviews as a means of understanding and interpreting existing literature. Work.

[33] Levac D, Colquhoun H, O’Brien KK (2010). Scoping studies: advancing the methodology. Implement Sci.

[34] Salim S. Work for Human Development. United Nations Development Programme; 2015.

[35] Miles MB, Huberman AM. Qualitative Data Analysis: An Expanded Sourcebook. Sage; 1994.

[36] United Nations Development Programme (UNDP). Human Development Report 2016: Human Development for Everyone. United Nations Development Programme; 2016.

[37] Lindskog BV (2014). Natural calamities and ‘the Big Migration’: challenges to the Mongolian health system in ‘the Age of the Market’. Glob Public Health.

[38] Kugel C, Zuroweste EL (2010). The state of health care services for mobile poor populations: history, current status, and future challenges. J Health Care Poor Underserved.

[39] Patterson J, Hovey DL (2000). Family-centered care for children with special health needs: Rhetoric or reality. Fam Syst Health.

[40] LeMay NV, Bocock PJ (2012). Building a national model for knowledge exchange in Malawi: findings from a health information needs assessment. J Health Commun.

[41] Sheiman IM, Shishkin SV (2010). Russian Health Care: New Challenges and New Objectives. Problems of Economic Transition.

[42] Forbes DA, Edge DS (2009). Canadian home care policy and practice in rural and remote settings: challenges and solutions. J Agromedicine.

[43] Thomason J, Kase P, Ndugwa N (2009). Working together to get back to basics--finding health system solutions. P N G Med J.

[44] Melo DG, Sequeiros J (2012). The challenges of incorporating genetic testing in the unified national health system in Brazil. Genet Test Mol Biomarkers.

[45] Al-Sharqi OZ, Abdullah MT (2013). “Diagnosing” Saudi health reforms: is NHIS the right “prescription”?. Int J Health Plann Manage.

[46] Chopra M, Lawn JE, Sanders D (2009). Achieving the health Millennium Development Goals for South Africa: challenges and priorities. Lancet.

[47] Warsame A, Handuleh J, Patel P (2016). Prioritization in Somali health system strengthening: a qualitative study. Int Health.

[48] Yip W, Hsiao WC (2008). The Chinese health system at a crossroads. Health Aff (Millwood).

[49] Cowan J, Greenberg Cowan J, Barnhart S (2013). A qualitative assessment of challenges to tuberculosis management and prevention in Northern Ethiopia. Int J Tuberc Lung Dis.

[50] Morhason-Bello IO, Odedina F, Rebbeck TR (2013). Challenges and opportunities in cancer control in Africa: a perspective from the African Organisation for Research and Training in Cancer. Lancet Oncol.

[51] Cristofalo M, Boutain D, Schraufnagel TJ, Bumgardner K, Zatzick D, Roy-Byrne PP (2009). Unmet need for mental health and addictions care in urban community health clinics: frontline provider accounts. Psychiatr Serv.

[52] Merican MI, Rohaizat Y, Haniza S (2004). Developing the Malaysian health system to meet the challenges of the future. Med J Malaysia.

[53] Morariu A (2014). The management of the human resources in the public health system: the complexity and the Euro-global socio-economic challenges. Rev Cercet Interv Soc.

[54] Peiro M, Barrubes J (2012). New context and old challenges in the healthcare system. Rev Esp Cardiol (Engl Ed).

[55] Ruff B, Mzimba M, Hendrie S, Broomberg J (2011). Reflections on health-care reforms in South Africa. J Public Health Policy.

[56] Davari M, Haycox A, Walley T (2012). The Iranian health insurance system; past experiences, present challenges and future strategies. Iran J Public Health.

[57] McGrail K, Zierler A, Ip I (2009). Getting what we pay for? The value-for-money challenge. Healthc Pap.

[58] Hanlon C, Luitel NP, Kathree T (2014). Challenges and opportunities for implementing integrated mental health care: a district level situation analysis from five low- and middle-income countries. PLoS One.

[59] Groman RF, Rubin KY (2013). Neurosurgical practice and health care reform: moving toward quality-based health care delivery. Neurosurg Focus.

[60] Halfon N, Conway PH (2013). The opportunities and challenges of a lifelong health system. N Engl J Med.

[61] Lehoux P, Miller FA, Daudelin G, Denis JL (2017). Providing Value to New Health Technology: The Early Contribution of Entrepreneurs, Investors, and Regulatory Agencies. Int J Health Policy Manag.

[62] Batayeh BG, Artzberger GH, Williams LDA (2018). Socially responsible innovation in health care: Cycles of actualization. Technol Soc.

[63] Lindley MC, Shen AK, Orenstein WA, Rodewald LE, Birkhead GS (2009). Financing the delivery of vaccines to children and adolescents: challenges to the current system. Pediatrics.

[64] Lewis S (2015). A system in name only--access, variation, and reform in Canada’s provinces. N Engl J Med.

[65] Jenkins R, Othieno C, Okeyo S, Aruwa J, Kingora J, Jenkins B (2013). Health system challenges to integration of mental health delivery in primary care in Kenya--perspectives of primary care health workers. BMC Health Serv Res.

[66] Greenhalgh T, Fahy N, Shaw S (2017). The bright elusive butterfly of value in health technology development: Comment on “Providing value to new health technology: the early contribution of entrepreneurs, investors, and regulatory agencies. ” Int J Health Policy Manag.

[67] Hamidi S, Shaban S, Mahate AA, Younis MZ (2014). Health insurance reform and the development of health insurance plans: the case of the Emirate of Abu Dhabi, UAE. J Health Care Finance.

[68] Nishtar S (2006). The Gateway Paper--proposed health reforms in Pakistan--interface considerations. J Pak Med Assoc.

[69] Yip WC, Hsiao WC, Chen W, Hu S, Ma J, Maynard A (2012). Early appraisal of China’s huge and complex health-care reforms. Lancet.

[70] Kerr A, Hill RL, Till C (2018). The limits of responsible innovation: Exploring care, vulnerability and precision medicine. Technol Soc.

[71] Lehoux P, Daudelin G, Williams-Jones B, Denis JL, Longo C (2014). How do business model and health technology design influence each other? Insights from a longitudinal case study of three academic spin-offs. Res Policy.

