# Supplementary material for: What Health System Challenges Should Responsible Innovation in Health Address? Insights From an International Scoping Review
Source: Int J Health Policy Manag. 2018 Nov 28;8(2):63–75. doi: 10.15171/ijhpm.2018.110 (PMC6462209; doi:10.15171/ijhpm.2018.110)
Supplement: Supplementary file 1 — contains Table S1. [file ijhpm-8-63-s001.pdf]

Supplementary File 1

Table S1. Examples of System-Level Challenges Along the HDI

**Low HDI**	**Medium HDI**	**High HDI**	**Very high HDI**
**Service delivery**			
"Inequities in healthcare treatment and access have important socioeconomic consequences" (Sub-Saharan Africa).[1]<br><br>"Re-orienting existing service delivery mechanisms from acute to chronic care, as well as ensuring equitable access to life-long ARV treatment, are often challenging for countries emerging from conflict, given the fragmented nature of the national health service system in the post-conflict	"To adapt the services that evolved towards provision of long term care for patients on ART, and to accommodate patients with other chronic illnesses such as hypertension, diabetes and cancer" (Zambia).[3]<br><br>"The withdrawal of the state may have widened the options for the more affluent, with the emergence of better services in some of the private facilities, but for the majority of the population this process has meant higher and higher out-	"Payments are dominated by outpatient pharmaceutical costs, and there is potentially significant unmet need in access due to shortages" (Belarus).[5]<br><br>"The changing epidemiology and demand patterns, in particular in urban areas, means that the services provided are no longer appropriate for the current and emerging needs" (Iran).[6]<br><br>"Rehabilitation, long-term and palliative care are not well developed as other parts of the health system which	"Increased use of health services has a direct impact on health spending" (Spain).[8]<br><br>"To effectively engage parents as full partners in all aspects of the planning and delivery of services and to achieve acceptance of mental health services" (US). [9]

Low HDI	Medium HDI	High HDI	Very high HDI
environment" (Burundi, Liberia, Mozambique, Rwanda, Sierra Leone and Uganda).[2]	of-pocket costs" (Syria).[4]	affects the system's overall efficiency. […] Mental health services are sorely lacking, and what is available is poorly integrated into the primary care system" (Armenia).[7]	
**Human resources**			
The "lack of appropriate medications" and "excess of certain medicines" creates confusion in "health workers' minds about whether they are expected to order medicines or not" when drug kits are not standardized (Kenya).[10]<br><br>"Political interference was singled out as a major reason for staff absenteeism, negligence to duties	"Maldistribution of staff and poor skills of many health personnel, which has compromised the ability to deliver key programmes, notably for HIV, tuberculosis, child health, mental health, and maternal health. Human resources have also been unevenly distributed between the public and private sectors, within the public sector" in favour of large, mostly urban-based medical	"The health system faces some basic structural problems" including a "lack sufficient human resources" (Mexico).[14]<br><br>"Human resources are not sufficient and are unequally distributed in the different geographical areas of the country" (Brazil).[15]<br><br>The inpatient system "remains poorly balanced, with an oversupply of capacity and staff in the capital often providing services to patients who would	The "demand for higher salaries for specialized medical staff" increased the cost of healthcare (Saudi Arabia).[16].<br><br>"A major contributing factor to this increasing home care human resource shortage is the limited public funding to home care" (Canada).[17]<br><br>When certified nursing assistants "enter a home to administer care, they must use whatever devices or equipment the patient owns," such as blood glucose meters or feeding pumps and thus have to

Low HDI	Medium HDI	High HDI	Very high HDI
and inability of the managers to take disciplinary action against influential staff members” (Pakistan).[11]	schools” (South Africa).[12]<br><br>“‘Woefully inadequate’, ‘skeletal’ and ‘acute shortages’ of mental health care professionals and funds dedicated to mental health” (Ghana).[13]	be more appropriately treated in day-care or outpatient settings” (Armenia).[7]	interact with unfamiliar devices, which “may increase the potential for usage errors” (US)[18]
**Leadership & governance**			
“Almost all participants expressed a desire to shift away from parallel systems and vertical programming, which is how many international agencies currently operate” (Myanmar).[19]<br><br>“The fact that after a decade of the existence of the scheme, coverage has remained largely	“Problems are still to be overcome by the health system, ranging from operational issues related to service delivery to strategic issues such as formulating an explicit privatisation policy, reducing fragmentation of, and aligning the external aid” (Tajikistan).[21]<br><br>“Poor systemic coordination and significant functional overlap with services	Rural doctors buy “expired and counterfeit drugs at low cost and sell them as valid products at higher prices” (China).[23]<br><br>“The list of compensated medicines is negotiated/ supplemented/ updated at too large intervals of time (several years) to the meet the needs of the citizens who find themselves unable to purchase the medication necessary to their	“Perverse incentives throughout the market-based system encouraging increased volume of services, emphasis on profits within a business model of care, lack of price controls, industry-friendly mechanisms for coverage and reimbursement decisions about new technologies, high administrative costs in our multi-payer financing system, aging of the population and

Low HDI	Medium HDI	High HDI	Very high HDI
negligible puts doubts on the commitments of the political class to the scheme and its ability to address the urgent issue of poor maternal health indices" (Nigeria).[20]	provided by private clinics and the government health center" (India).[22]	conditions, and are forced to resort to hospital services even more expensive for the public health budget" (Romania).[24]<br><br>"Prices of up to 16 times higher than the international reference prices were found among private retail pharmacies" and "an overall lack of pricing policy and suboptimal public sector drug procurement" (Malaysia).[25]	increasing application of new technologies" (US).[26]<br><br>"Health care investments have been poorly coordinated and guided by local economic interests rather than by the health needs of the population" (Hungary).[27]
**Infrastructure & supplies**			
"Basic health facilities and staff housing are in poor shape in most parts of the country, and there is no systematic asset maintenance and replacement program" (Papua New Guinea).[28]	"Inadequate architecture" described as "a source of inefficiency" (India).[30]<br><br>India shares "a problem common to other emerging and high-income economies: the	"There are rising consumer demands and expectations for expanding technology and high cost medical care due to improved standards of living, changing disease rising patterns and	"Several rural physicians in our sample explained the lack of medical infrastructure as problematic and the geographical isolation as formidable barriers to providing care" (US).[35]<br><br>Infrastructural barriers people with disabilities

Low HDI	Medium HDI	High HDI	Very high HDI
"Lack of certain essentials" such as gloves, gauze or syringes and patients "had to buy these themselves" (Kenya).[10]<br><br>"No radiotherapy facilities" although "as many as 50% of all patients with cancer would be expected to benefit from radiotherapy" (sub-Saharan Africa).[1]<br><br>"Unavailability or intermittent supply of electricity" and "essential drugs with no alternatives" "missing from the market for several years" (Pakistan).[29]	unsustainable prices of cancer drugs" (India).[31]<br><br>"Despite considerable investment in public infrastructure, poorer, rural areas generally have more frequent stock-outs of key medical supplies, less access to diagnostic test facilities, emergency transport, and various clinical equipment, and less basic infrastructure" (South Africa).[32]<br><br>"Limited availability of diagnostic equipment and medicines to treat the range of NCDs" [non-communicable diseases] (Zambia).[3]	demographic shifts" (Malaysia).[33]<br><br>"Increased income allows Chinese people to afford more expensive medical tests and treatment, and enhanced health awareness makes them willing to pay more money for health care, especially in urban areas. New medical tests and treatments are frequently requested by patients during their hospital visits, even though these tests and treatments are much more expensive and are not covered by medical insurance" (China).[34]	face when seeking to obtain care and services, including "stairs with no ramp, inaccessible restrooms, and no sign language interpreter capability" (US).[36]
**Knowledge & Information systems**			
The "need for the development of appropriate advocacy	"Records maintained are not adequately used as tools for	"The main weak points of the current information system are	Need for "embracing technological advances that will reduce the

Low HDI	Medium HDI	High HDI	Very high HDI
strategies," for "innovative ways of knowledge-sharing" and for the mobilization of key stakeholders to "effectively communicate evidence" (Pakistan).[29]<br><br>"Neither clients' needs nor the workload in each clinic could be adequately captured by the health management information system" (Kenya).[10]	enhancing service quality" (India).[37]<br><br>"Pharmaceutical companies had easy access to doctors for influencing their practice through personal periodic visits by company representatives, sponsoring of continuing medical education activities and provision of medical literature to doctors" (India).[22]	poor analyzability and stratification. These weaknesses have caused the information system not to be used for decision making" (Iran).[6]<br><br>"Poor communication between different health care delivery systems in the Mexican system aggravates problems associated with the complex referral system. Even within vertical programs, like that for tuberculosis, private doctors, the Secretarıa de Salud (SSA), and the IMSS systems do not readily share information about patients" (Mexico).[38]	reliance on face- to-face doctor interaction as the principal mode for health requirements. While aspects of these are already in place a coordinated approach is still elusive" (Australia).[39]<br><br>"Parents of infants and toddlers may also have limited experience navigating service systems and require additional support in order to understand that their voices actually count in setting service priorities and designing treatment plans" (US).[9]

References

1. Morhason-Bello IO, Odedina F, Rebbeck TR, et al. Challenges and opportunities in cancer control in Africa: a perspective from the African Organisation for Research and Training in Cancer. *Lancet Oncol.* 2013;14(4):e142-151. doi:10.1016/s1470-2045(12)70482-5

2. John-Langba J. National Health Systems and Unmet Need for Antiretroviral Medication and HIV/AIDS-Related Healthcare in African Countries Emerging from Conflict. *International Peacekeeping.* 2013;20(4):427-438. doi:10.1080/13533312.2013.846134

3. Aantjes CJ, Quinlan TK, Bunders JF. Practicalities and challenges in re-orienting the health system in Zambia for treating chronic conditions. *BMC Health Serv Res.* 2014;14:295. doi:10.1186/1472-6963-14-295

4. Ahmad B, Fouad FM, Elias M, Zaman S, Phillimore P, Maziak W. Health system challenges for the management of cardiovascular disease and diabetes: an empirical qualitative study from Syria. *Int J Public Health.* 2015;60 Suppl 1:S55-62. doi:10.1007/s00038-014-0594-2

5. Richardson E, Malakhova I, Novik I, Famenka A. Belarus: health system review. *Health Syst Transit.* 2013;15(5):1-118.

6. Moghadam MN, Sadeghi V, Parva S. Weaknesses and challenges of primary healthcare system in Iran: a review. *Int J Health Plann Manage.* 2012;27(2):e121-131. doi:10.1002/hpm.1105

7. Richardson E. Armenia: health system review. *Health Syst Transit.* 2013;15(4):1-99.

8. Peiro M, Barrubes J. New context and old challenges in the healthcare system. *Rev Esp Cardiol (Engl Ed).* 2012;65(7):651-655. doi:10.1016/j.recesp.2012.02.019

9. Finello KM, Poulsen MK. Unique System of Care Issues and Challenges in Serving Children Under Age 3 and their Families. *American Journal of Community Psychology.* 2012;49(3-4):417-429. doi:http://dx.doi.org/10.1007/s10464-011-9458-6

10. Jenkins R, Othieno C, Okeyo S, Aruwa J, Kingora J, Jenkins B. Health system challenges to integration of mental health delivery in primary care in Kenya--perspectives of primary care health workers. *BMC Health Serv Res.* 2013;13:368. doi:10.1186/1472-6963-13-368

11. Mir AM, Gull S. Countdown to 2015: A case study of maternal and child health service delivery challenges in five districts of Punjab. *Journal of the Pakistan Medical Association.* 2012;62(12):1308-1313.

12. Coovadia H, Jewkes R, Barron P, Sanders D, McIntyre D. Health in South Africa 1 The health and health system of South Africa: historical roots of current public health challenges. *Lancet.* 2009;374(9692):817-834. doi:10.1016/s0140-6736(09)60951-x

13. Doku V, Ofori-Atta A, Akpalu B, Osei A, Read U, Cooper S. Stakeholders' perceptions of the main challenges facing Ghana's mental health care system: A qualitative analysis. *International Journal of Culture and Mental Health.* 2011;4(1):8-22. doi:10.1080/17542863.2010.503038

14. Laurell AC. Health system reform in Mexico: a critical review. *Int J Health Serv.* 2007;37(3):515-535.

15. Mateus MD, Mari JJ, Delgado PG, et al. The mental health system in Brazil: Policies and future challenges. *Int J Ment Health Syst.* 2008;2(1):12. doi:10.1186/1752-4458-2-12

16. Al-Sharqi OZ, Abdullah MT. "Diagnosing" Saudi health reforms: is NHIS the right "prescription"? *Int J Health Plann Manage.* 2013;28(4):308-319. doi:10.1002/hpm.2148

17. Forbes DA, Edge DS. Canadian home care policy and practice in rural and remote settings: challenges and solutions. *Journal of agromedicine.* 2009;14(2):119-124. doi:http://dx.doi.org/10.1080/10599240902724135

18. McBride SE, Beer JM, Mitzner TL, Rogers WA. Challenges for home health care providers: A needs assessment. *Physical & Occupational Therapy in Geriatrics.* 2011;29(1):5-22. doi:10.3109/02703181.2011.552170

19. Risso-Gill I, McKee M, Coker R, Piot P, Legido-Quigley H. Health system strengthening in Myanmar during political reforms: perspectives from international agencies. *Health Policy Plan.* 2014;29(4):466-474. doi:10.1093/heapol/czt037

20. Ebeigbe PN. Reducing maternal mortality in Nigeria: the need for urgent changes in financing for maternal health in the Nigerian health system. *Niger Postgrad Med J.* 2013;20(2):148-153.

21. Mirzoev TN, Green AT, Newell JN. Progress towards health reform in Tajikistan. *J Health Organ Manag.* 2007;21(6):495-505. doi:10.1108/14777260710834292

22. Bhojani U, Devedasan N, Mishra A, De Henauw S, Kolsteren P, Criel B. Health system challenges in organizing quality diabetes care for urban poor in South India. *PLoS One.* 2014;9(9):e106522. doi:10.1371/journal.pone.0106522

23. Yip W, Hsiao WC. The Chinese health system at a crossroads. *Health Aff (Millwood).* 2008;27(2):460-468. doi:10.1377/hlthaff.27.2.460

24. Morariu A. The Management of the Human Resources in the Public Health System: The Complexity and the Euro-Global Socio-Economic Challenges. *Revista De Cercetare Si Interventie Sociala.* 2014;44:266-278.

25. Mohd-Tahir NA, Paraidathathu T, Li SC. Quality use of medicine in a developing economy: Measures to overcome challenges in the Malaysian healthcare system. *SAGE Open Med.* 2015;3:2050312115596864. doi:10.1177/2050312115596864

26. Geyman JP. Cost-Sharing under Consumer-Driven Health Care Will Not Reform U.S. Health Care. *Journal of Law, Medicine and Ethics.* 2012;40(3):574-581. doi:http://dx.doi.org/10.1111/j.1748-720X.2012.00690.x

27. Gaal P, Szigeti S, Csere M, Gaskins M, Panteli D. Hungary health system review. *Health Syst Transit.* 2011;13(5):1-266.

28. Thomason J, Kase P, Ndugwa N. Working together to get back to basics--finding health system solutions. *P N G Med J.* 2009;52(3-4):114-129.

29. Nishtar S. The Gateway Paper--proposed health reforms in Pakistan--interface considerations. *J Pak Med Assoc.* 2006;56(12 Suppl 4):S78-93.

30. Kumar JR. Role of public health systems in the present health scenario: key challenges. *Indian J Public Health.* 2013;57(3):133-137. doi:10.4103/0019-557x.119808

31. Pramesh CS, Badwe RA, Borthakur BB, et al. Delivery of affordable and equitable cancer care in India. *Lancet Oncol.* 2014;15(6):e223-233. doi:10.1016/s1470-2045(14)70117-2

32. Gilson L, McIntyre D. Post-apartheid challenges: household access and use of health care in South Africa. *Int J Health Serv.* 2007;37(4):673-691.

33. Merican MI, bin Yon R. Health care reform and changes: the Malaysian experience. *Asia Pac J Public Health.* 2002;14(1):17-22.

34. Wang C, Rao K, Wu S, Liu Q. Health care in China: improvement, challenges, and reform. *Chest.* 2013;143(2):524-531. doi:10.1378/chest.12-1839

35. Putzer GJ, Koro-Ljungberg M, Duncan RP. Critical challenges and impediments affecting rural physicians during a public health emergency. *Disaster medicine and public health preparedness.* 2012;6(4):342-348.

36. Leigh IW, Powers L, Vash C, Nettles R. Survey of Psychological Services to Clients With Disabilities: The Need for Awareness. *Rehabilitation Psychology.* 2004;49(1):48-54. doi:10.1037/0090-5550.49.1.48

37. Agarwal S. Improving urban newborn health: Challenges and the way forward. *Journal of Neonatology.* 2009;23(3):208-216.

38. Molina RL, Palazuelos D. Navigating and circumventing a fragmented health system: the patient's pathway in the Sierra Madre Region of Chiapas, Mexico. *Med Anthropol Q.* 2014;28(1):23-43. doi:10.1111/maq.12071

39. Iyngkaran P, Harris M, Ilton M, et al. Implementing guideline based heart failure care in the Northern Territory: challenges and solutions. *Heart Lung Circ.* 2014;23(5):391-406. doi:10.1016/j.hlc.2013.12.005
